# Calculations of helium separation via uniform pores of stanene-based membranes

**DOI:** 10.3762/bjnano.6.256

**Published:** 2015-12-23

**Authors:** Guoping Gao, Yan Jiao, Yalong Jiao, Fengxian Ma, Liangzhi Kou, Aijun Du

**Affiliations:** 1 School of Chemistry, Physics and Mechanical Engineering, Queensland University of Technology, Garden Point Campus, Brisbane QLD 4001, Australia,; 2 School of Chemical Engineering, University of Adelaide, Adelaide SA 5005, Australia

**Keywords:** fluorination, gas purification, honeycomb lattice

## Abstract

The development of low energy cost membranes to separate He from noble gas mixtures is highly desired. In this work, we studied He purification using recently experimentally realized, two-dimensional stanene (2D Sn) and decorated 2D Sn (SnH and SnF) honeycomb lattices by density functional theory calculations. To increase the permeability of noble gases through pristine 2D Sn at room temperature (298 K), two practical strategies (i.e., the application of strain and functionalization) are proposed. With their high concentration of large pores, 2D Sn-based membrane materials demonstrate excellent helium purification and can serve as a superior membrane over traditionally used, porous materials. In addition, the separation performance of these 2D Sn-based membrane materials can be significantly tuned by application of strain to optimize the He purification properties by taking both diffusion and selectivity into account. Our results are the first calculations of He separation in a defect-free honeycomb lattice, highlighting new interesting materials for helium separation for future experimental validation.

## Introduction

With many outstanding properties such as low density, low boiling point, low solubility, and high thermal conductivity and inertness, helium finds extensive application in cryogenic science [1], arc welding processes [2], and leak detection [3]. Although it is the second most abundant element on earth, the helium concentration in the atmosphere is very low (about 5.2 ppm) [4]. Only in some natural gas fields is the helium concentration high enough for commercial separation by an energy-intensive process, such as fraction distillation. The development of a low energy cost membrane to separate helium is therefore highly desired.

In recent years, various two-dimensional materials have been developed [5–6] and are widely used as membranes for gas separation [7–10]. The pore size is the main determinant of a membrane with high permeability and selectivity for helium purification. Traditional 2D membranes such as graphene and silicene are known to be impermeable to helium due to their small pores. In order to enhance the helium separation performance, defects are introduced in graphene and silicone [11–13]. However, obtaining precise and controllable defect sizes on graphene and silicene remains an experimental challenge [10]. Other single atom thick, 2D membranes, such as pristine g-C_3_N_4_ and graphdiyne, have been reported for hydrogen purification but are not suitable for helium separation due to their large pores [8]. Therefore, the development of new, pristine, two-dimension materials with an ideal pore size is desired for helium separation.

As a new member of the family of layered materials following graphene, silicene and germanene, 2D stanene has been recently successfully fabricated by molecular beam epitaxy [14]. 2D stanene possesses a graphene-like honeycomb lattice, but its lattice constant is dramatically larger than that of graphene and silicene. Theoretical calculations have shown that decorating 2D Sn with chemical functional groups such as H and F can further increase the lattice constant [15]. Therefore, the pores of pristine and functionalized stanene are much larger than those of pristine graphene and silicene, which allows for promising application of stanene-based materials for helium separation. In this paper, we demonstrate that 2D Sn-based membranes with a high concentration of uniform pores are excellent candidates for helium purification. Most interestingly, the 2D Sn-based materials can be further strain-engineered to achieve improved He separation performance by taking both diffusion and selectivity into account.

## Computational Method

Density functional theory (DFT) calculations were carried out using the Vienna ab initio simulation package (VASP) [16–17]. The exchange-correlation interactions were described by a generalized gradient approximation (GGA) [18] with the Perdew–Burke–Ernzerhof (PBE) functional [19]. Spin-polarization and a damped van der Waals correction in Grimme’s scheme [20] were included in all the calculations. The cut-off energy for plane waves was set at 500 eV, and the convergence criteria for residual force and energy on each atom during structure relaxation were set to 0.005 eV/Å and 10^−5^ eV, respectively. The vacuum space was more than 20 Å, which was enough to avoid the interaction between periodic images. The climbing image nudged elastic band (NEB) was used to find the saddle points and minimum energy paths. The 2 × 2 unit cells of 2D Sn, SnH and SnF were used as the model for NEB calculations. The initial, transition, and final geometry of He penetrating through 2D Sn are shown in Supporting Information File 1, Figure S1. The Brillouin zone was sampled with the Monkhorst−Pack mesh [21] with a k-point of 6 × 6 × 1 grid in reciprocal space during geometry optimization and NEB calculations.

The most stable state of noble gas and membrane is defined as the initial state (IS). The penetration barrier is calculated by:

[1]
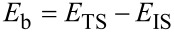


where the *E*_TS_ and *E*_IS_ are the total energy of the saddle point and initial state, respectively.

The diffusion rate, *k*, is estimated based on the penetration barrier, *E*_b_, through the Arrhenius equation, as in previous works [12,22]:

[2]
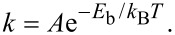


Here, the diffusion prefactor, *A*, is set to 10^11^ s^−1^ [12] and *k*_B_ and *T* are the Boltzmann constant and the absolute temperature, respectively. The rate of selectivity, *S*, is the diffusion rate of He, *k*(He), divided by that of the gas, *k*(gas), as shown in the following equation:

[3]
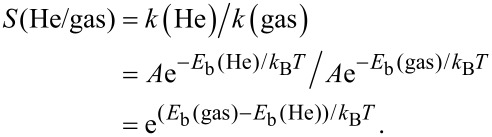


## Results and Discussion

**Figure 1:**
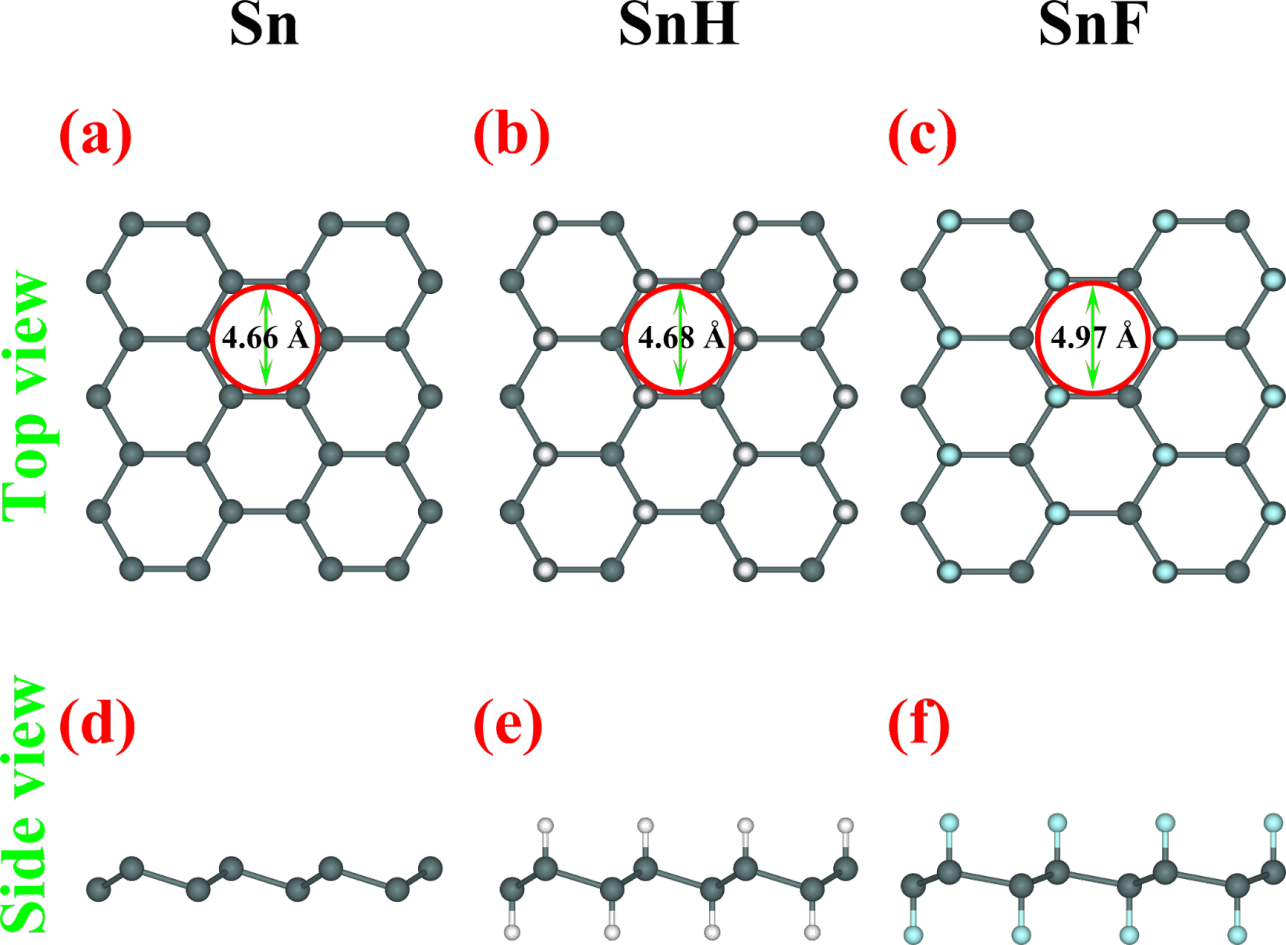
The geometrical structures of Sn, SnH, and SnF lattices from top (a–c) and side (d–f) views. Color code: grey, Sn; white, H; light green, F.

Figure 1 presents the structures of stanene, hydrogenated stanene (SnH), and fluorinated stanene (SnF) studied in this work. The fully relaxed lattice constants of the three stanene-based systems are calculated to be 4.66 Å, 4.68 Å, and 4.97 Å, respectively, which are in agreement with previous results [15]. Their stability is further confirmed by phonopy calculations, as shown in Supporting Information File 1, Figure S2. No image frequency is found for any of the three membranes, indicating dynamic stability. The pores are normally and uniformly distributed on surface of the three membrane systems. In a honeycomb lattice, the pore diameters of the three membranes are equal to the lattice constants. Additionally, the 2D Sn, SnH, and SnF structures possess a concentration of pores significantly higher than that of porous graphene or silicene [11–13].

**Figure 2:**
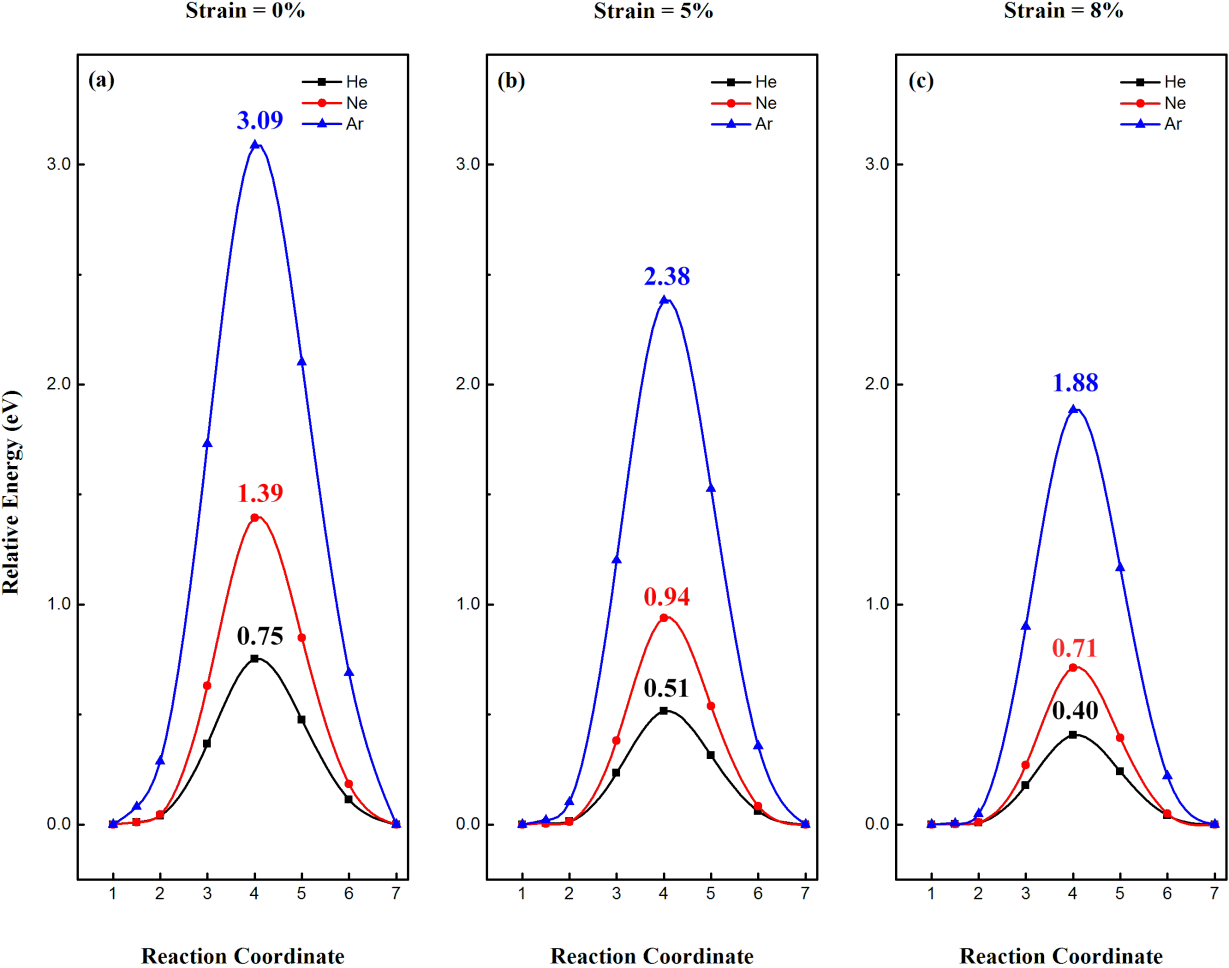
Minimum pathway for noble gases (He, Ne and Ar) penetrating through 2D Sn lattice under (a) 0%, (b) 5% and (c) 8% strain.

Next, we investigated the minimum pathway for noble gases (He, Ne and Ar) penetrating through 2D Sn, SnH, and SnF using the NEB method. The effect of strain on the penetration of noble gases were investigated as well. Figure 2 shows the minimum pathways for noble gases passing through the pristine 2D Sn in the presence of 0%, 5% and 8% strain. Without strain, the penetration barrier for He, Ne and Ar passing through the 2D Sn are 0.75, 1.39 and 3.09 eV, respectively. Clearly, the selectivity is high, but the penetration barrier for He through pristine 2D Sn is quite high, indicating a low permeability at room temperature. Since the pore size is critical for gas penetration, a small tensile strain applied to the 2D Sn is expected to increase the penetration efficiency of noble gases. Under 5% strain, the penetration barrier for He, Ne and Ar is significantly decreased to 0.51, 0.94 and 2.38 eV, respectively. When the strain on 2D Sn is further increased to 8%, the penetration barrier for He, Ne and Ar is further decreased to 0.40, 0.71 and 1.88 eV, respectively. The penetration barrier of He is smaller than the threshold barrier [11] for gas penetration (about 0.5 eV), while the penetration barrier for the other two noble gases is larger than the threshold barrier. Therefore, the helium separation performance of 2D Sn can be significantly improved by inducing a small strain.

**Figure 3:**
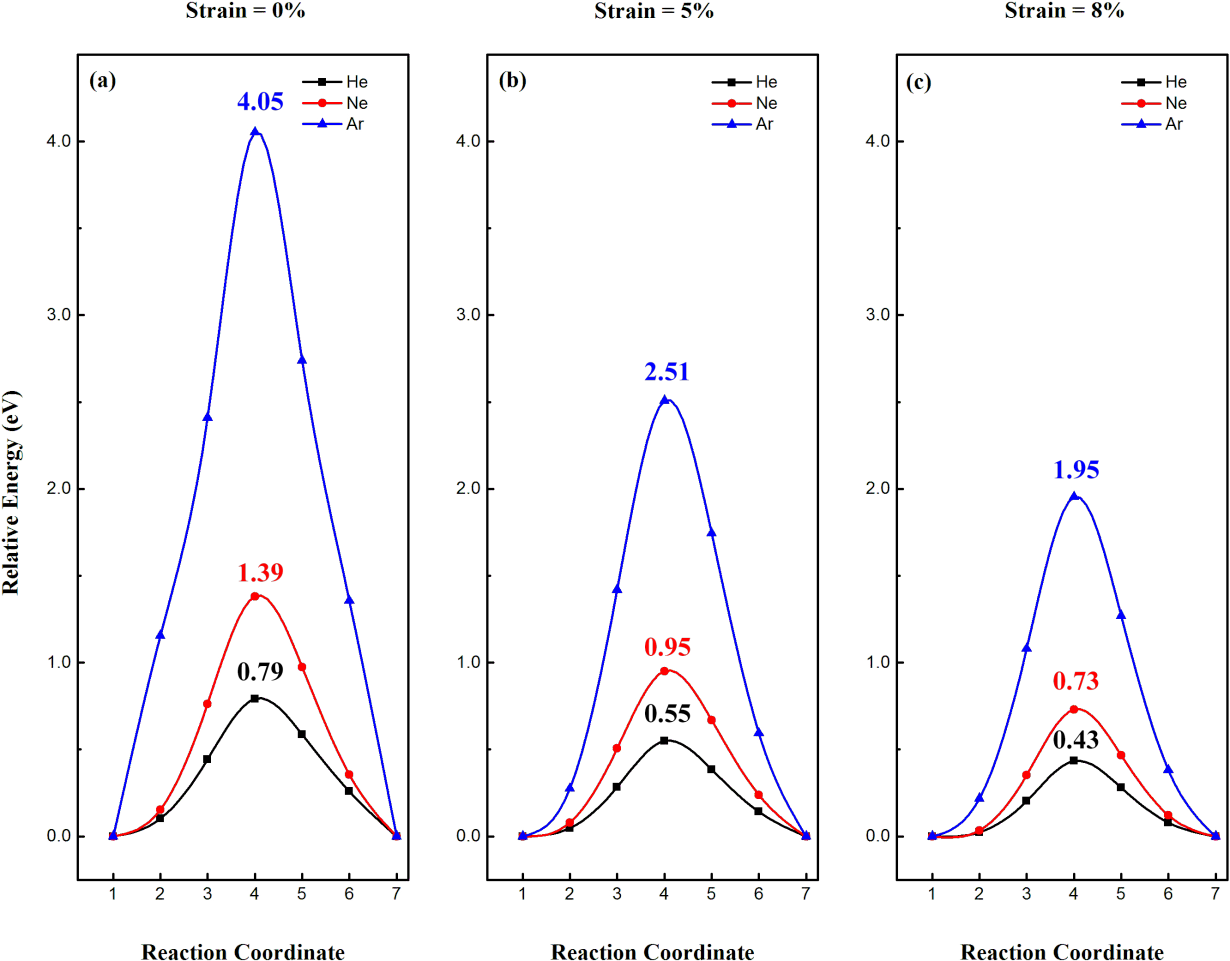
Minimum pathway for noble gases (He, Ne and Ar) passing through a 2D SnH lattice under (a) 0%, (b) 5% and (c) 8% strain.

When the 2D Sn surface is functionalized with hydrogen, the lattice constant of 2D SnH lattices is slightly larger (0.02 Å) than that of 2D Sn, and as a result, the gas penetration behavior will basically be similar to that of the stanene layer. The minimum pathway for noble gases (He, Ne and Ar) passing through 2D SnH under different strains are shown in Figure 3. Without strain, the penetration barrier for He, Ne and Ar passing through the 2D SnH is 0.79, 1.39 and 4.05 eV, respectively. It should be noted that these values are slightly larger than that of 2D Sn. Under 5 and 8% strain, the penetration barrier of noble gases through the 2D SnH is also slightly larger than the corresponding value for 2D Sn. Even though the lattice constant of 2D SnH is larger than that of 2D Sn, the penetration efficiency of noble gases is not as good as that of 2D Sn. Therefore, the hydrogen functionalization of 2D Sn results in the degradation of the noble gas permeability due to the steric effect caused by hydrogen decoration.

**Figure 4:**
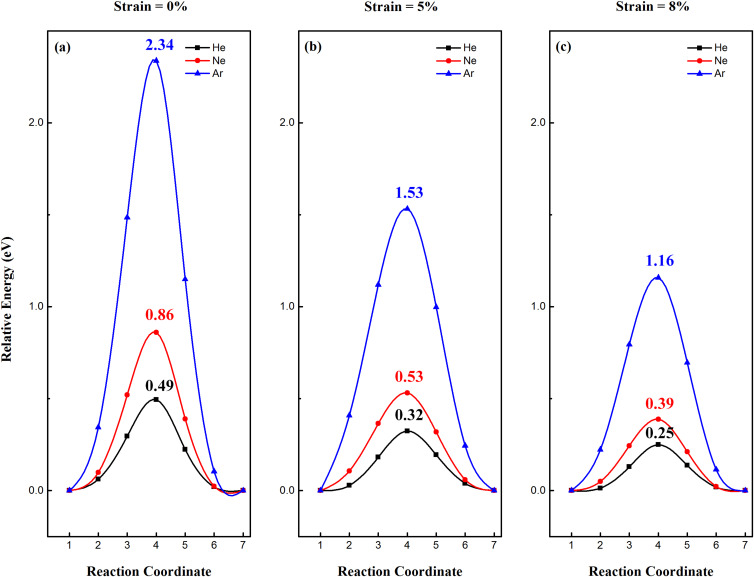
Minimum pathway for noble gases (He, Ne and Ar) passing through a 2D SnF lattice under (a) 0%, (b) 5% and (c) 8% strain.

It is expected that the stanene functionalized with fluorine will be a good candidate for He separation due to a lower buckling and larger lattice constant. The lattice constant of 2D SnF is much larger than that of 2D Sn, which is expected to significantly facilitate the gas penetration. The minimum pathways for noble gases (He, Ne and Ar) passing through the 2D SnF under different strain are shown in Figure 4. In the absence of strain, the penetration barrier for He, Ne and Ar passing through the 2D SnF lattice is calculated to be 0.49, 0.86 and 2.34 eV, respectively. These values are much smaller than the penetration barrier for noble gases passing through 2D Sn and SnH. The penetration barrier of helium through 2D SnF is smaller than the threshold barrier (0.5 eV) and is comparable to that of previous results [11–13]. Furthermore, under moderate strain, the penetration barrier of noble gases through 2D SnF can be further reduced. For example, the penetration barrier of He, Ne and Ar is reduced to 0.25, 0.39 and 1.16 eV, respectively, under a strain of 8%. Thus, the 2D Sn-based membrane materials can be applied as potential membranes for helium separation, where the penetration properties can be tuned by application of a small amount of strain.

**Figure 5:**
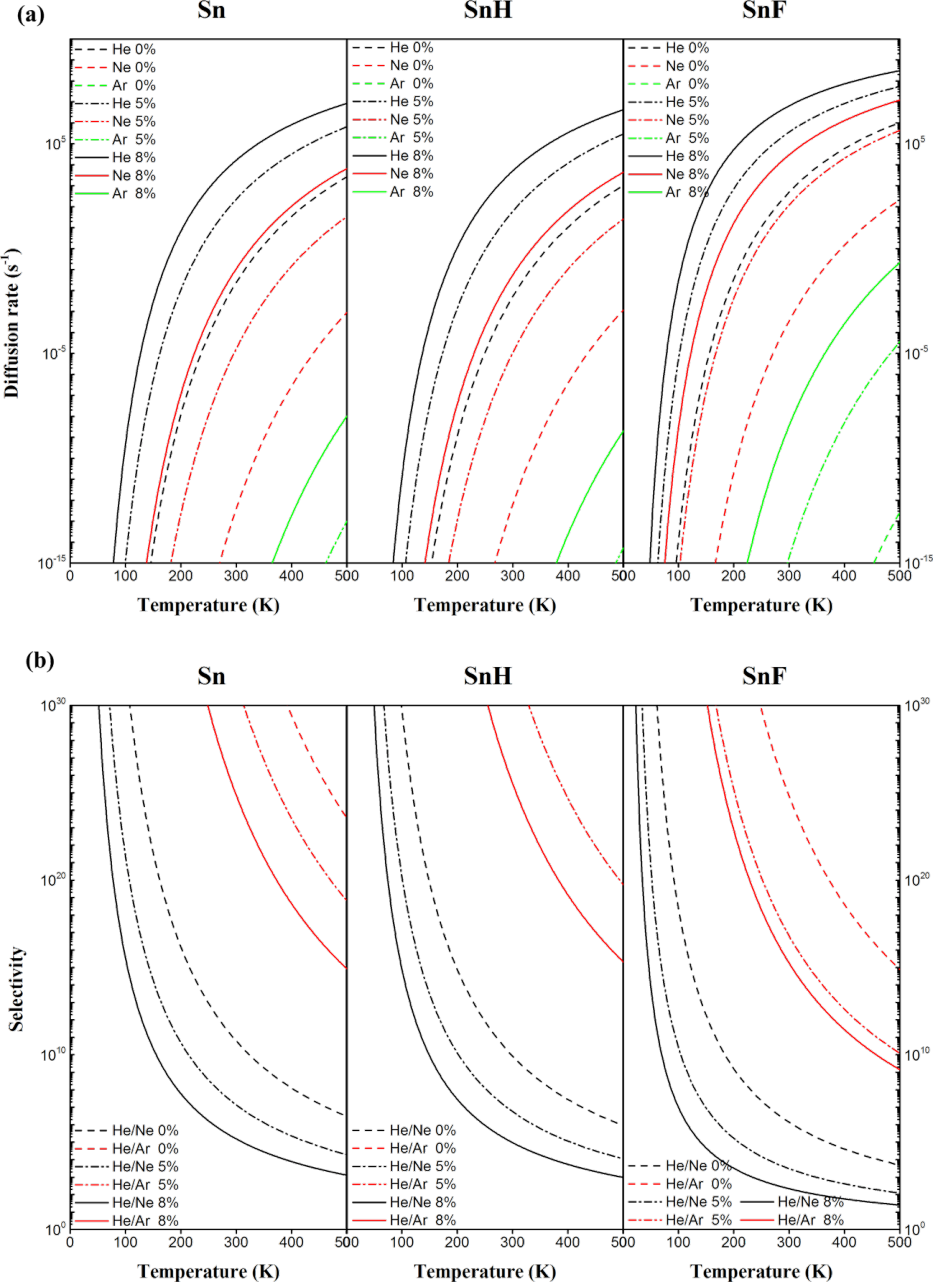
Diffusion rate (a) and selectivity (b) for noble gases (He, Ne and Ar) penetrating through 2D Sn, SnH, and SnF lattices under different strains.

To quantitatively examine the helium separation efficiency on a 2D Sn-based membrane material, the noble gas diffusion rate and selectivity of He from Ne/Ar were calculated based on the Arrhenius equation (see Equation 2 and Equation 3). The noble gas diffusion rate and the selectivity of He/Ne and He/Ar are plotted as a function of temperature in Figure 5a,b, respectively. The major challenges of helium purification mainly come from the diffusion rate of He and the selectivity of He/Ne and He/Ar. Without strain, the gas diffusion rate in 2D Sn, SnH and SnF lattices is 2.0 × 10^−2^, 4.2 × 10^−3^, and 4.4 × 10^2^ respectively, and the selectivity of He/Ne separation is 7.3 × 10^10^, 9.7 × 10^9^, and 1.5 × 10^6^, respectively, at room temperature. Among the three studied membranes, only the 2D SnF membrane is He permeable. As the strain increases, the diffusion rate increases at the sacrifice of selectivity. Under 8% strain, the gas diffusion rate in 2D Sn, SnH and SnF is 1.5 × 10^4^, 4.6 × 10^3^, and 6.1 × 10^6^, respectively, and the selectivity of He/Ne is 1.7 × 10^5^, 1.0 × 10^5^, and 2.3 × 10^2^, respectively, at room temperature. Clearly, better performance can be achieved under moderate strain. Therefore, we can tune the strain of 2D Sn membranes to achieve the desired He separation performance by taking both permeability and selectivity into account.

## Conclusion

In summary, the helium separation performance through new, experimentally realized, pristine and H/F-decorated 2D Sn lattices under different amounts of strain were systematically investigated by DFT calculations. At room temperature, the pristine 2D Sn is impermeable for noble gases. To increase the diffusion rate of noble gases, two practical strategies were proposed: stretch and fluorination. With a high concentration of uniform pores, 2D Sn-based materials exhibited excellent performance for He purification and can serve as a superior membrane as compared to traditional porous materials. In addition, these 2D Sn-based membrane materials can be significantly tuned using strain to optimize He separation performance by taking both diffusion and selectivity into account.

## Supporting Information

File 1 The phonon dispersion spectrum and stress–strain curves of Sn, SnH and SnF lattices.
