# Supplementary material for: Calculations of helium separation via uniform pores of stanene-based membranes
Source: Beilstein J Nanotechnol. 2015 Dec 23;6:2470–6. doi: 10.3762/bjnano.6.256 (PMC4734409; doi:10.3762/bjnano.6.256)
Supplement: File 1 — The phonon dispersion spectrum and stress–strain curves of Sn, SnH and SnF lattices. [file Beilstein_J_Nanotechnol-06-2470-s001.pdf]

Supporting Information

for

Helium separation via uniform pores of stanene-based membranes

Guoping Gao[1], Yan Jiao[2], Yalong Jiao[1], Fengxian Ma[1], Liangzhi Kou[1] and Aijun Du[1,]*

Address: [1]School of Chemistry, Physics and Mechanical Engineering, Queensland University of Technology, Garden Point Campus, Brisbane QLD 4001, Australia, and [2]School of Chemical Engineering, University of Adelaide, Adelaide SA 5005, Australia

Email: Aijun Du - aijun.du@qut.edu.au

* Corresponding author

The phonon dispersion spectrum and stress–strain curves of Sn, SnH and SnF lattices

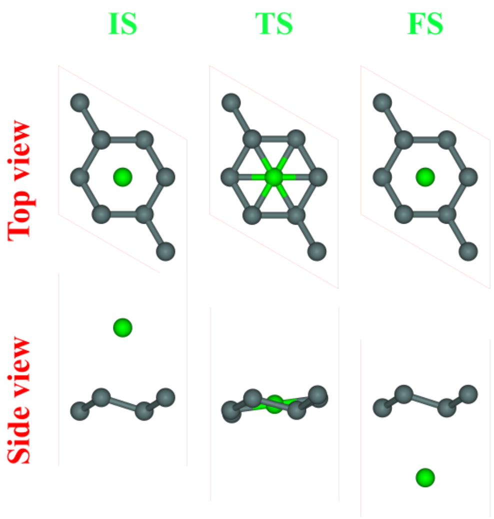

Figure S1: The initial, transition, and finial geometries of He penetrating through 2D Sn. Color code: grey, Sn; green, He.

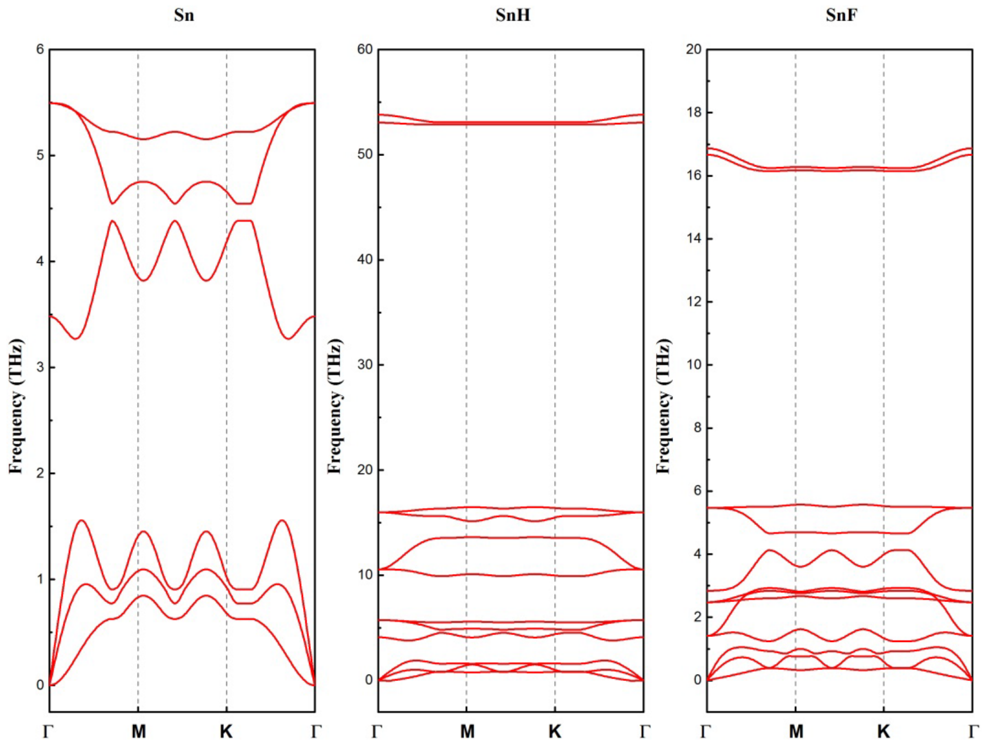

Figure S2: The phonon dispersion spectrum of Sn, SnH and SnF.

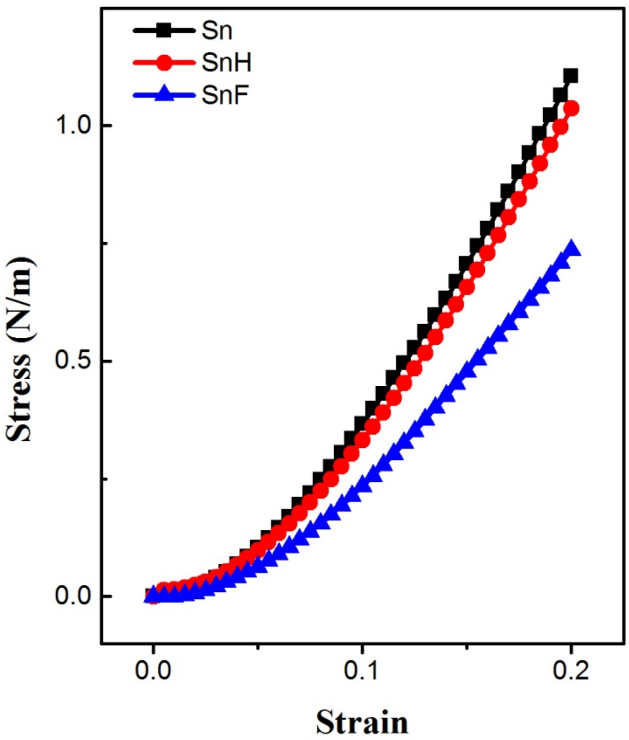

Figure S3: The stress–strain curves for Sn, SnH and SnF membranes.
